# The management of pneumothorax in patients with anorexia nervosa: A case report and review of the literature

**DOI:** 10.1186/1754-9493-4-1

**Published:** 2010-02-01

**Authors:** Walter L Biffl, Vignesh Narayanan, Jennifer L Gaudiani, Philip S Mehler

**Affiliations:** 1 Department of Surgery, Denver Health Medical Center, Denver, Colorado, USA; 2 Department of Patient Safety and Quality, Denver Health Medical Center, Denver, Colorado, USA; 3 Department of Medicine, Denver Health Medical Center, Denver, Colorado, USA

## Abstract

Of the many body systems adversely affected by severe anorexia nervosa (AN), the pulmonary system is relatively spared. However, in the face of severe malnutrition of AN, the lung may undergo architectural changes that adversely affect its integrity and healing capacity. We report herein a case of a pneumothorax in a patient with severe AN, in which standard approaches to manage the pneumothorax were unsuccessful. Despite prolonged tube thoracostomy drainage, and subsequent thoracoscopic pleuredesis, the patient continued to have an air leak and non-resolution of her pneumothorax. We review the literature and discuss alternative approaches in this patient population.

## Introduction

Anorexia nervosa (AN) can be associated with a litany of medical complications. A recent review cited adverse effects on the gastrointestinal, cardiovascular, hematologic, endocrine, renal, neurologic and dermatologic systems [1]. The pulmonary system may be affected as well. Although rare, there are descriptions of spontaneous pneumothorax [2,3], spontaneous pneumomediastinum [4,5], and emphysema-like changes [6] in patients with AN. We report herein a case of prolonged air leak following a pneumothorax in a patient with AN, and discuss management strategies in light of potential pulmonary pathology unique to AN.

## Case Report

**Figure 1:**
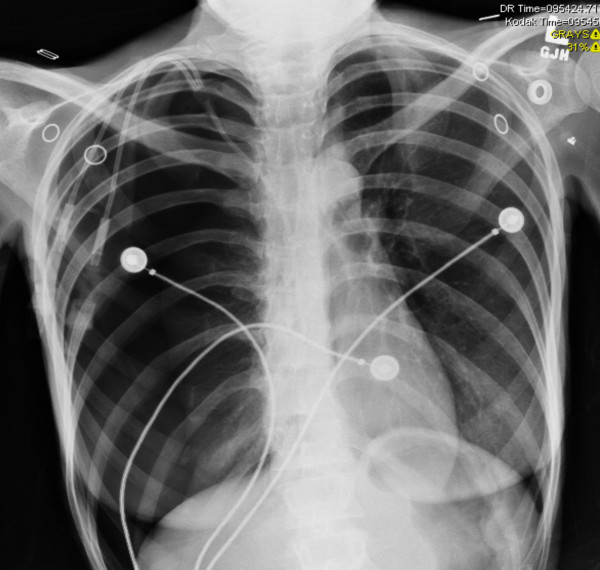
**Chest x-ray demonstrating large right pneumothorax on the day after placement of the central venous catheter**.

A 26-year-old female with severe AN was admitted to our A.C.U.T.E. (Acute Comprehensive Urgent Treatment for Eating disorders) medical stabilization program for monitored weight restoration to enable eventual transfer to an inpatient eating disorder program. At the time of hospitalization the patient was depressed and had a body mass index (BMI) of 10.3 kg/m^2^. She displayed hypothermia (34.2°C), a blood pressure of 72/40 mmHg and heart rate of 36 bpm. Her skin was notable for prominent xerosis, and she had poor venous access. Laboratory testing revealed leukopenia and a marked elevation of her blood urea-nitrogen and serum aminotransferases (AST and ALT > 1,000 u/L). Her serum total protein and albumin levels were 6.9 and 4.4 g/dL, both within the normal range. She also quickly developed refeeding hypophosphatemia [7]. Because of the need for frequent blood draws and intravenous fluids to treat her marked dehydration and hypotension, a 7-French double-lumen Hohn catheter was inserted by an interventional radiologist into her right internal jugular vein via an anterior cervical approach using ultrasound localization and fluoroscopic guidance. The procedure went smoothly and without apparent complication, and a post-procedure chest x-ray was unremarkable. However, the next day an x-ray obtained for unrelated reasons demonstrated a sizable right pneumothorax (Figure 1). Although the patient was asymptomatic, a 10-French pigtail thoracostomy catheter was inserted at the bedside and attached to 20 cm H_2_O suction to evacuate the pneumothorax and facilitate lung re-expansion.

After one week, the patient appeared to have complete re-expansion of the lung on x-ray, but the pneumothorax returned a day later. Moreover, she appeared to develop a spontaneous left pneumothorax. The left pneumothorax resolved spontaneously, but she had a persistent right-sided pneumothorax and air leak from the tube. The tube entry site and pleural drainage system were examined and re-dressed on two occasions, but the air leak persisted, suggesting a probable alveolar-pleural fistula. Since she remained asymptomatic from a pulmonary standpoint, nonoperative management was continued to avoid interference with her weight restoration. Unfortunately, the air leak remained unchanged, and after 13 days it was decided that video-assisted thoracoscopic surgery (VATS) was indicated and offered the best opportunity for definitive resolution [8]. On thoracoscopic exploration there was no visible pathology of the lung, and despite submerging the lung and insufflating air under pressure, no air leak could be demonstrated. Mechanical pleurodesis was performed circumferentially around the upper half of the pleural space, and two new thoracostomy tubes were placed. One chest tube was removed a day later, but the patient continued to have an air leak and persistent pneumothorax. The tube entry site and pleural drainage system were once again examined, but the air leak persisted. A few days later, an image-guided pigtail tube was placed into her pleural space, and the other chest tube was removed. On the twelfth postoperative day a Heimlich valve was connected to the pigtail pleural tube to allow the patient greater mobility and possible discharge. Eight days later- 33 days after it was first discovered- the pneumothorax had resolved, and the pigtail tube was pulled. The patient has had no recurrence and has gained 35 pounds (BMI 17.6).

## Discussion

Anorexia nervosa (AN) is the purest form of human malnutrition, in that it is not secondary to an inflammatory, infectious, or neoplastic process, and affects multiple organ systems [9]. A number of cases of spontaneous pneumothorax and pneumomediastinum have been reported in patients with AN, but the pathophysiology of these events remains unclear [2-5]. In animal models, prolonged starvation leads to decreases in total lung protein content, connective tissue, hydroxyproline and elastin [10]. Consequently, the architecture may be weakened and may be more susceptible to injury [3].

Patients with severe AN require frequent blood draws during the early stages of their refeeding program, as well as a need for intravenous fluids, blood products and occasionally parenteral nutrition [11]. However, venous access for these therapies may be quite limited in patients who are severely malnourished. In addition, the discomfort and annoyance to the patient of daily venipuncture may interfere with the therapeutic treatment plan and medical stabilization. Therefore, in terms of the management of this patient, the central venous catheter was considered a necessary intervention, and appropriate precautions were used to minimize the risk of complications. A relatively high anterior approach to the internal jugular vein was selected, and ultrasound was used to guide the insertion.

In the current case, although the immediate post-procedure film did not demonstrate a pneumothorax, the temporal association suggests this was likely an iatrogenic, and not a spontaneous, pneumothorax. Furthermore, on thoracoscopic examination, the right lung appeared normal, without evidence of apical bullae or any other significant pathology. On the other hand, the prolonged air leak suggests that she had some underlying abnormality. Malnutrition is a well-known impediment to normal wound healing. This patient had normal serum protein and albumin levels at the time of admission. However, malnutrition was assumed based on her overall condition and pattern of weight loss. Indeed, we have found that conventional markers of nutritional status such as serum albumin may not correlate with the severity of AN, and could be paradoxically normal even in very advanced stages of this disease [12]. Moreover, as Coxson and colleagues [6] described, patients with AN may develop architectural changes and decreased surfactant production. This could result in a lung that heals poorly and will not seal itself. The fact that no abnormality- specifically, no hole- was found in the lung at the time of thoracoscopic examination, does not necessarily mean that a hole did not exist. Indeed, the fact that the air leak continued suggests that a perforation was missed. It is possible that the lack of inflammation or exudate at the site of injury made it more difficult to detect. It is unlikely that further imaging (e.g., CT scan) would have directed our efforts, as there was no gross pathology present on visual inspection of the lung. Similarly, in the absence of visible pathology, lung resection would have been difficult to justify- particularly in the face of compromised wound healing.

Tube thoracostomy allows re-expansion and resolution of pneumothoraces, but it may take variable amounts of time for air leaks to seal. Persistent air leak is accepted as an indication for VATS [8]. In this case, VATS was deferred based on concern that post-procedural pain and the need for narcotic pain medications would impede her feeding progress. In general, the low morbidity and typical course (i.e., eating a regular diet soon after the procedure) of VATS would make this a minor concern, but patients with AN can be very sensitive to changes in their course, and may be easily derailed from their nutritional plan. In retrospect, the procedure was of dubious benefit. Although most healthy individuals do well with removal of one chest tube in the early postoperative period, it may be prudent to leave two tubes in place in the patient with AN. Furthermore, it is possible that higher levels of suction would have been successful in promoting lung expansion and sealing following the pleuredesis procedure. This should be considered in future cases. An alternative strategy, as proposed by Cerfolio [13], would have been to switch from suction or water seal drainage to a Heimlich valve earlier in the course. In fact, in consultation with a pulmonologist, it was decided that this would be a reasonable step toward patient discharge. We suggest that this strategy is safe and allows full mobility and earlier discharge.

## Conclusions

In sum, the malnutrition of AN is associated with pulmonary changes that may predispose to spontaneous pneumothorax or persistent air leak. Whether these changes are reversible with refeeding, and how long it takes to reestablish normal pulmonary physiology are not currently known. However, a prolonged alveolar-pleural fistula may be anticipated in these patients during the severe stage of their AN. Based on our experience, this problem may be managed effectively with a Heimlich valve to expedite the patient's transition to the next stage of their recovery from AN.

## Abbreviations List

ALT: Alanine aminotransferase; AN: Anorexia nervosa; AST: Aspartate aminotransferase; BMI: Body mass index; VATS: Video-assisted thoracoscopic surgery

## Consent

Written informed consent was obtained from the patient for publication of this case report and accompanying images. A copy of the written consent is available for review by the Editor-in-Chief of this Journal.

## Competing interests

The authors declare that they have no competing interests.

## Authors' contributions

WLB, VN, JLG, and PSM have all made substantial contributions to the drafting of the manuscript and critical revision, and all have given final approval of the version to be published.
